# Dysbiosis patterns during re-induction/salvage versus induction chemotherapy for acute leukemia

**DOI:** 10.1038/s41598-019-42652-6

**Published:** 2019-04-15

**Authors:** Armin Rashidi, Thomas Kaiser, Robin Shields-Cutler, Carolyn Graiziger, Shernan G. Holtan, Tauseef Ur Rehman, Justin Wasko, Daniel J. Weisdorf, Gary Dunny, Alexander Khoruts, Christopher Staley

**Affiliations:** 1 Division of Hematology, Oncology, and Transplantation, Department of Medicine, University of Minnesota, Minneapolis, MN USA; 2 Department of Surgery, University of Minnesota, Minneapolis, MN USA; 3 BioTechnology Institute, University of Minnesota, St. Paul, MN USA; 4 Department of Biology, Macalester College, St. Paul, Minnesota USA; 5 Division of Gastroenterology, Hepatology, and Nutrition, Department of Medicine, University of Minnesota, Minneapolis, MN USA; 6 Department of Pharmacy, University of Minnesota Medical Center, Minneapolis, MN USA; 7 Department of Microbiology and Immunology, University of Minnesota, Minneapolis, MN USA

## Abstract

Acute leukemia (AL) patients undergoing intensive induction chemotherapy develop severe gut dysbiosis, placing them at heightened risk for infectious complications. Some AL patients will undergo “repeat therapy” (re-induction or salvage) due to persistent or relapsed disease. We hypothesized that prior injury to the microbiome during induction may influence dysbiosis patterns during repeat therapy. To test this hypothesis, we analyzed the bacterial microbiome profiles of thrice-weekly stool samples from 20 intensively treated AL patients (first induction: 13, repeat therapy: 7) by 16S rRNA sequencing. In mixed-effects modeling, repeat therapy was a significant predictor of *Enterococcus* expansion (*P* = 0.006), independently of antibiotic exposure, disease type, feeding mode, and week of chemotherapy. Bayesian analysis of longitudinal data demonstrated larger departures of microbial communities from the pre-chemotherapy baseline during repeat therapy compared to induction. This increased ecosystem instability during repeat therapy possibly impairs colonization resistance and increases vulnerability to *Enterococcus* outgrowth. Microbiota restoration therapies at the end of induction or before starting subsequent therapy warrant investigation.

## Introduction

Intensive chemotherapy for acute leukemia (AL) is typically accompanied by prolonged exposure to multiple antibiotics over a ~1-month inpatient stay, which constitutes a major ecological disruption to the intestinal microbiota^1–3^. The resulting dysbiosis leads to overgrowth of pathobionts enriched in antibiotic resistance genes and adept at translocation via the compromised gut barrier^4^. Despite widespread use of prophylactic antibiotics during intensive anti-leukemia chemotherapy, enteric bacteria are still responsible for >40% of all bloodstream infections (BSIs) and the causative organism is multidrug-resistant in ~20% of cases^5^. In addition, dysbiosis is the leading risk factor for *Clostridium difficile* infection (CDI), occurring in ~10% of AL patients^6^. Although treatment-related mortality has decreased in recent years^7^, infection remains a significant cause of morbidity and mortality and a barrier to success in curative-intent, anti-leukemia chemotherapy.

Patients with persistent disease after one cycle of induction and those relapsing after an initial remission commonly require intensive “repeat therapy” (re-induction or salvage). The clinical severity of gut barrier damage during intensive repeat therapy is thought to be comparable to the initial treatment, but can be higher if repeat therapy is started shortly after the initial treatment, before complete recovery from prior damage. However, although repeat therapy patients are at particularly high risk for infectious complications, current standard supportive care, including anti-microbial therapy, is largely independent of the treatment phase. We hypothesized that the experience of prior intensive chemotherapy may influence dysbiosis patterns during repeat therapy. This would have implications for supportive care optimization, including potential restorative microbiota therapies. To address this question, we compared gut dysbiosis patterns in intensively treated AL patients according to treatment phase.

## Methods

We analyzed longitudinal stool samples (n = 207) from 20 unique AL patients undergoing intensive inpatient chemotherapy. Patients with acute promyelocytic leukemia were not included. The initial treatment for medically fit adult patients with non-M3 acute myeloid leukemia (AML) is most commonly “7 + 3”, the combination of an anthracycline (3 days) and cytarabine (7 days)^8^. Moderate to severe mucotoxicity is common (~25% of patients) with ~10% of patients requiring parenteral nutrition^8,9^. Regimens used for repeat therapy differ in agents and duration of administration but are generally short-duration (5–10 days) combined regimens with comparable toxicity profile^10,11^. At our center, we use MEC (mitoxantrone, etoposide, and cytarabine for 5 days)^12^ for fit patients and Clo/Ara-C (clofarabine for 5 days and low-dose cytarabine for 10 days)^13^ for the less fit. The frequency and severity of mucositis with MEC or Clo/Ara-C are comparable to 7 + 3^14,15^. There are several standard chemotherapy induction protocols for ALL, all based on multi-agent regimens with varying risks of mucotoxicty. Most of those regimens are thought to be somewhat less mucotoxic than intensive AML protocols, though formal comparisons have not been made. At our center, we use the PETHEMA^16^ and GRAAL^17^ regimens for induction in ALL patients.

We collected the first sample before or on day 1 of chemotherapy and continued thrice-weekly collections until day 30 or discharge, whichever occurred first. Samples were stored at −80 °C. Levofloxacin was administered for antibacterial prophylaxis until neutrophil recovery, and cefepime as empiric antibiotic for neutropenic fever. Variations in antibiotic choice were at the discretion of treating physicians. Parenteral nutrition was used only if oral intake was considered inadequate. The University of Minnesota Institutional Review Board approved the protocol, and the study was performed in accordance with the Declaration of Helsinki. Informed consent was obtained from all participants.

DNA was extracted from stool using the DNeasy PowerSoil kit (Qiagen, Hilden, Germany). The V4 hypervariable region (515F/806R primer set^18^) of the 16S rRNA gene was amplified and paired-end sequenced (2 × 300 nucleotides) on the Illumina MiSeq platform (Illumina, Inc., San Diego, CA) at the University of Minnesota Genomics Center^19^. Negative (sterile water) controls were included and did not produce amplicons. Sequence data were processed in mothur^20^, as described previously^21^. Sequences were trimmed to 170 nt to remove low-quality regions at the 3′-end and paired-end joined using the fastq.join script^21^. Sequences were quality filtered over a window of 50 nt at a quality threshold of 35, and those with ≥2 mismatches to primer sequences, homopolymers ≥8 nt, or ambiguous bases were removed. High quality sequences were aligned against the SILVA database version 132^22,23^ for downstream processing and subjected to a 2% pre-cluster to remove likely sequence errors^24^. Chimeric sequences were identified and removed using UCHIME version 4.2.40^25^. Operational taxonomic units (OTUs) were classified at 97% sequence similarity using the furthest-neighbor algorithm and taxonomic classifications were made against the version 16 release from the Ribosomal Database Project^26^.

Samples were normalized to 9500 sequence reads/sample. Alpha diversity was calculated using the Shannon index^27^. Comparison of longitudinal samples to baseline (beta diversity) was done using SourceTracker (ST) version 0.9.8^28^. For ST analysis, the baseline sample for each patient was used as “source”, and subsequent samples were used as “sink” samples. ST uses a Bayesian algorithm that leverages the information contained in taxa distributions to infer taxa attributions, thus the percentages reported reflect the estimated overlap in community composition between baseline and subsequent samples (*i*.*e*., similarity to baseline). This information cannot be derived from more conventional beta diversity indices such as the Bray-Curtis distance^29^. When sequences in the sink sample could not be unambiguously assigned to the baseline sample, they were assigned to an “unknown” source (newly introduced taxa or due to statistical ambiguity because of low abundances) and interpreted as divergent from baseline. Linear discriminant analysis (LDA) of effect sizes (LEfSe) was used to determine differentially abundant OTUs in the two groups; these OTUs were then classified to genera^30^. Species-level assignment of highly differential (LDA score ≥ 4.0) OTUs was done using BLAST^31^.

To evaluate the independent association between treatment phase and taxa, we applied generalized linear mixed-effects modelling using the glmmTMB package in R, where models containing fixed and random effects are fitted using maximum likelihood estimation via Template Model Builder (TMB). We defined the model in Wilkinson notation^32^ as Taxon ~ Treatment phase + Week of chemotherapy + Antibiotic class i (i = 1–4) + total parenteral nutrition (TPN) + Disease + (1|Patient), where taxon (relative abundance) is the dependent variable. The fixed-effects covariates (all categorical) are treatment phase (0 if induction, 1 if repeat therapy; forced in all models), week of chemotherapy (baseline or 0 vs. 1 vs. 2 vs. 3 vs. 4), TPN (1 if used within 7 days before sample, 0 otherwise), disease (ALL vs. AML), antibiotic (1 if used within 7 days before sample, 0 otherwise). The random-effects covariate is patient number. We used week, rather than day, of chemotherapy to permit categorization because the dynamics of taxa relative abundance over time was non-monotonic in many cases. We considered four classes of antibiotics, most commonly used in our patients: fluoroquinolones, third (or higher) generation cephalosporins, anti-anaerobic antibiotics (piperacillin-tazobactam, carbapenems, metronidazole, and clindamycin), and vancomycin. We applied a backward stepwise selection algorithm, starting with the complete model and removing variables with the weakest (and non-significant) association with outcome until an optimal model with the lowest Akaike and Bayesian information criterion indices was reached. We used a Beta response distribution after adding a constant (10^−4^) to 0’s and subtracting 10^−4^ from 1’s to have a non-inclusive (0,1) boundary for relative abundances. We used a false discovery rate-adjusted *P* value of 0.01 to define statistical significance in analyzing the 5 and 10 most abundant phyla and genera, respectively.

Significant taxa in generalized linear mixed modelling were further analyzed with the *permuspliner* function of SplinectomeR, a permutation-based package in R that uses weighted local polynomials (loess splines) to test for group differences in longitudinal data^33^. This method is less sensitive to the limitations of using aggregate data over time. We performed 999 permutations. Finally, we used the *sliding_spliner* function of SplinectomeR to determine whether the groups are more significantly different in specific segments of time. This non-permutation-based technique divides the time axis to 100 segments and finds segments with larger contributions to the overall intergroup difference over time for a given taxon.

To evaluate the independent association between treatment phase and alpha diversity, we applied linear mixed-effects modeling using the lmer package in R and maximum likelihood estimation. We defined the model as Shannon index ~ Treatment phase + Week of chemotherapy + Antibiotic class + TPN + Disease + (1|Patient), using similar model selection approach and definitions as those detailed above. All statistical analyses were performed in R 3.4 (R Foundation for Statistical Computing, Vienna, Austria).

## Results

We studied 13 induction therapy and 7 repeat therapy patients (Table 1), who provided 133 and 74 samples, respectively. Sixteen patients had AML (9 induction therapy and 7 repeat therapy; 167 samples) and four had ALL (4 induction therapy and no repeat therapy; 40 samples). Among AML cases, 4 had myelodysplastic changes, 2 were therapy-related, and 1 was secondary to myelodysplastic syndrome. According to the 2017 European LeukemiaNet classification system^34^, 5 AML cases were favorable-risk, 6 were intermediate-risk, and 5 were adverse-risk (including a complex karyotype in 4 patients and complex monosomal karyotype in 1). All ALL cases were B-cell and high-risk (Ph-positive in 2 cases and Ph-like in 2). The median time from the most recent intensive chemotherapy in repeat therapy patients was 200 days. Prior intensive therapy in this group included the following: 7 + 3 (4 patients), HyperCVAD (1 patient), and 7 + 3 followed by high-dose cytarabine (2 patients). Of the 13 and 7 patients receiving induction and repeat therapy, 9 (69%) and 4 (57%) achieved a complete remission, respectively.

**Table 1:** Baseline patient, disease, and treatment characteristics.

	Induction chemotherapy (n = 13)	Repeat therapy (n = 7)
**Gender**, **n (%)**		
Male	5 (38)	4 (57)
Female	8 (62)	3 (43)
*Age, median (range)*	53 (22–74) years	52 (22–68) years
**Disease**, **n (%)**		
AML	9 (69)	7 (100)
ALL	4 (31)	0
**Chemotherapy**, **n (%)**		
7 + 3	9 (69)	0
GRAAL	2 (15)	0
PETHEMA	2 (15)	0
MEC	0	5 (71)
Clo/Ara-C	0	2 (29)

7 + 3: Idarubicin + Cytarabine; ALL: Acute lymphoblastic leukemia; AML: Acute myeloid leukemia; Clo/Ara-C: Clofarabine + Cytarabine; MEC: Mitoxantrone + Etoposide + Cytarabine.

**Figure 1:**
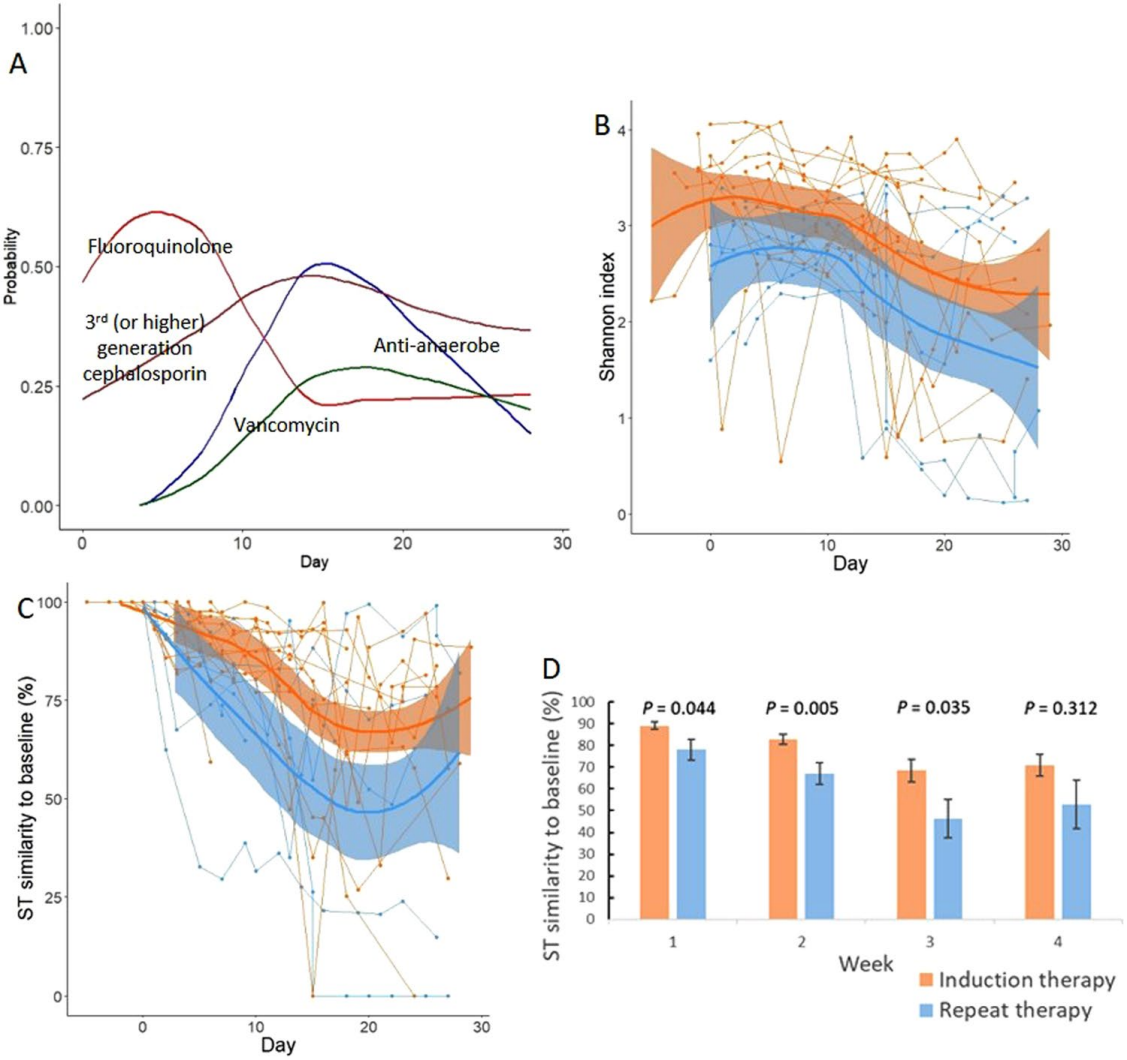
Antibacterial antibiotic use, diversity and stability of microbiota during induction and repeat therapy. Patterns of use for anti-anaerobic antibiotics, vancomycin, fluoroquinolones, and third (or higher)-generation cephalosporins are shown in (**A**), where the y axis shows the probability of antibiotic exposure. Panel (B) shows the Shannon diversity index. Panel (C) shows SourceTracker (ST) similarity to baseline for longitudinal samples. Spider charts in (**B**,**C**) compare samples collected during induction therapy (orange) vs. repeat therapy (blue) and include Loess splines with 95% confidence bands. Panel (D) shows a weekly comparison between the groups for ST similarity to baseline. Bar charts show mean of indices in samples collected in each week and standard error. *P* values are from a Mann-Whitney test.

Antibiotic exposure was intense, with 104 antibiotic doses (all types) and 43 anti-bacterial antibiotic doses per 30 patient-days; for antibiotics given >1 dose/day, only 1 dose/day was considered (Fig. 1A). Exposure to fluoroquinolones, anti-anaerobic antibiotics, third (or higher) generation cephalosporins, and vancomycin occurred in 11, 7, 10, and 7 of the 13 induction patients, respectively. These numbers were 7, 7, 4, and 5 in the 7 repeat therapy patients, respectively. During their inpatient stay, 18 (90%), 5 (25%), and 4 (20%) patients developed neutropenic fever, BSI, and CDI, respectively, and 5 (25%; 3 patients during induction and 2 during repeat therapy) received TPN. The isolated organisms from blood cultures were *Streptococcus mitis* (3 patients), *Strep*. *sanguinis* (1 patient), methicillin-resistant *Staphylococcus aureus* (1 patient), and vancomycin-resistant *Enterococcus faecium* (VRE; 1 patient). *Enterococcus* achieved a relative abundance of >10% in the two stool samples collected before VRE BSI.

A mean estimated Good’s coverage of 99.37 ± 0.03% was observed. Microbial diversity declined markedly with time (Fig. 1B), with Shannon indices reaching levels lower than even those we have reported in patients with multiply recurrent CDI prior to fecal microbiota transplantation^35^. Although repeat therapy samples had lower diversity, the difference did not reach statistical significance in linear mixed-effects modeling (*P* = 0.09). In contrast, recent use of TPN (*P* < 10^−4^), anti-anaerobic antibiotics (*P* < 10^−8^), or vancomycin (*P* < 10^−6^) was associated with lower diversity. Disease type (AML vs. ALL) was not independently associated with diversity.

We hypothesized that the experience of prior chemotherapy (*e*.*g*., outpatient-to-inpatient transition, chemotherapy, nutritional changes, and antibiotic exposures) may have a lasting effect on microbial ecosystems. One such detrimental effect may be diminished microbial ecosystem stability at the time of initiation of repeat therapy. As a surrogate for stability, we measured the similarity of microbiota in longitudinal samples throughout induction or repeat therapy to the baseline sample collected before initiating the corresponding treatment phase. In SourceTracker analysis, repeat therapy samples collected in weeks 1 through 3 showed less similarity to their baseline sample compared to the similarity of induction samples to their corresponding baseline sample (*P* < 0.05, Fig. 1C,D). Greater intestinal microbial ecosystem displacement in repeat therapy patients suggests loss of ecosystem stability relative to that seen in patients undergoing induction therapy.

**Figure 2:**
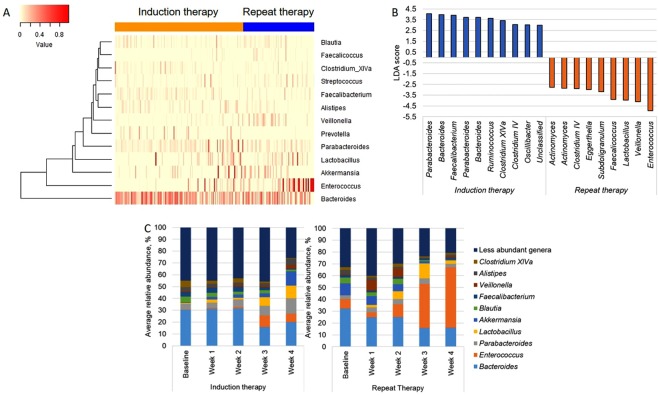
Composition of microbial communities during induction and repeat therapy. (**A**) Heat map showing genera relative abundances. (**B**) Linear discriminant analysis (LDA) plot highlighting differentially abundant taxa in induction therapy vs. repeat therapy samples. (**C**) Progressive expansion of *Enterococcus* (more prominent in repeat therapy samples). Only the 10 most abundant genera (averaged across all samples) are shown; the remaining taxa are combined as “less abundant genera”.

**Figure 3:**
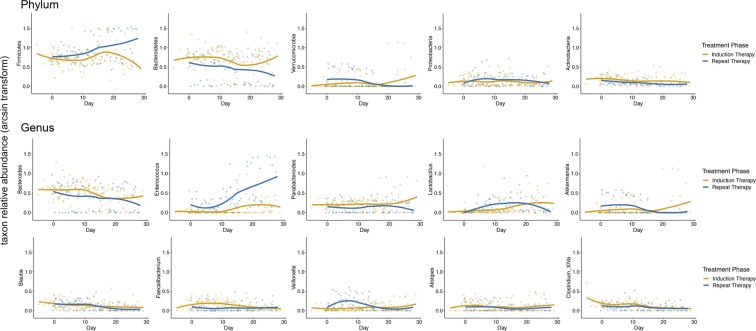
Fitted splines for induction vs. repeat therapy groups. Only the 5 most abundant phyla and 10 most abundant genera are shown. The y axis shows the relative abundance of each taxon after arcsine transformation for better visualization.

**Figure 4:**
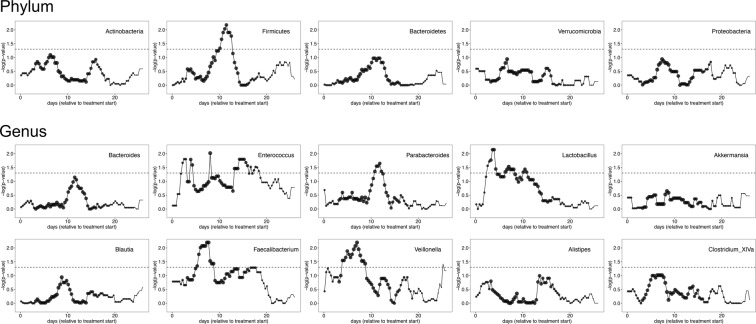
Statistical significance of the comparison between induction vs. repeat therapy groups over time. Only the 5 most abundant phyla and 10 most abundant genera are shown. The y axis shows the -log(*P*) from Mann-Whitney tests comparing the group splines at each interval. Dotted line indicates *P* = 0.05. The number of patients with data at a given interval is used to scale the data point size. 100 intervals are used.

One major consequence of diminished ecosystem stability is greater ease of invasion and expansion of previously rare taxa. Therefore, we evaluated whether the composition of microbial communities during intensive chemotherapy depends on treatment phase (Fig. 2A). When samples were analyzed in aggregate, the three most differentially abundant taxa were one *Enterococcus* OTU (LDA score: −4.95, *P* = 10^−11^) and one *Veillonella* OTU (*P* = 0.006) in repeat therapy samples, and one *Parabacteroides* OTU (classified as *Parabacteroides distasonis*; LDA score: 4.05, *P* = 3 × 10^−4^) in induction samples (Fig. 2B). In addition, repeat therapy samples experienced a progressive expansion of *Enterococcus* with time (Fig. 2C). Comparison of the two groups for longitudinal differences using splines fitted to *Enterococcus* relative abundance was significant (*P* = 0.027). In mixed-effects models (Table 2), *Enterococcus* was the only taxon independently associated with treatment phase (FDR-adjusted *P* = 0.006; Fig. 3). Other significant predictors of *Enterococcus* expansion were the use of TPN (*P* = 0.004) and anti-anaerobic antibiotics (*P* < 10^−6^). Finally, we evaluated whether inter-group differences during specific segments of time had a greater contribution to the overall difference in *Enterococcus* relative abundance between the groups. Considering day 0–14 and 15–28 intervals, the groups were different at several timepoints in both intervals. Figure 4 shows the unadjusted *P* values over time for the 5 most abundant phyla and 10 most abundant genera. Other taxa with significant differences between the groups in specific segments of time included the phylum Firmicutes and genera *Parabacteroides*, *Lactobacillus*, *Faecalibacterium*, and *Veillonella*, all in the interval between days 0–14, when patients tend to have highest levels of toxicity from chemotherapy.

**Table 2:** Mixed-effects modeling of the association between treatment phase and taxa relative abundance.

Phylum	Factor	β	*P*	q	Genus	Factor	β	*P*	q
Firmicutes	Rx	0.39	0.011	0.05	*Bacteroides*	Rx	−0.55	0.199	0.33
	TPN	0.99	<0.01			TPN	−1.84	<0.01	
	AA	1.17	<0.01			Vanc	−1.17	<0.01	
	CPN3+	−0.48	<0.01						
Bacteroidetes	Rx	−0.71	0.063	0.16	*Enterococcus*	Rx	1.14	<0.001	0.006
	TPN	−1.53	<0.01			TPN	1.11	<0.01	
	AA	−0.72	<0.01			FQN	−0.52	<0.01	
	CPN3+	0.45	<0.01			Vanc	0.93	<0.01	
	Vanc	−0.84	<0.01						
Verrucomicrobia	Rx	0.3	0.448	0.52	*Parabacteroides*	Rx	−0.58	0.142	0.27
	Vanc	−0.41	0.046			TPN	−0.99	0.028	
						FQN	−0.36	0.031	
						AA	−0.69	<0.01	
Proteobacteria	Rx	0.21	0.535	0.53	*Lactobacillus*	Rx	0.37	0.085	0.18
						CPN3+	0.38	0.043	
						Vanc	0.61	<0.01	
Actinobacteria	Rx	−0.16	0.52	0.53	*Akkermansia*	Rx	0.29	0.451	0.52
	ALL	1.31	<0.01						
	FQN	0.44	<0.01						
	Vanc	−0.52	<0.01						
					*Blautia*	Rx	−0.34	0.247	0.34
						CPN3+	−0.65	<0.01	
						Vanc	−1.18	<0.01	
					*Faecalibacterium*	Rx	−1.11	0.004	0.03
						Vanc	−1.09	<0.01	
						Week 1	1.53	<0.01	
						Week 2	1.52	<0.01	
						Week 3	1.37	<0.01	
						Week 4	1.09	0.021	
					*Veillonella*	Rx	0.65	0.032	0.1
						AA	−0.6	<0.01	
					*Alistipes*	Rx	−0.54	0.223	0.33
						Vanc	−0.72	<0.01	
					*Clostridium cluster XIVa*	Rx	−0.57	0.014	0.05
						Vanc	−0.9	<0.01	
						Week 1	−0.56	0.074	
						Week 2	−0.46	0.143	
						Week 3	−0.98	<0.01	
						Week 4	−1.11	<0.01	

β shows the regression coefficient. q represents adjusted *P* (false discovery rate method) and is shown for the main effect (treatment phase) only. AA: Anti-anaerobic antibiotic; ALL: Acute lymphoblastic leukemia (vs. acute myeloid leukemia); CPN3+ : Third (or higher) generation cephalosporin; FQN: Fluoroquinolone; Rx: Treatment phase (repeat therapy vs. induction); TPN: Total parenteral nutrition; Vanc: Vancomycin; Week: week of chemotherapy (vs. before treatment initiation).

## Discussion

Although the development of dysbiosis in AL patients has been reported^1,2^, whether the patterns of dysbiosis differ in different phases of therapy is not known. This knowledge has critical implications for potential microbiota therapeutics because AL patients often receive multiple cycles of intensive chemotherapy and are repeatedly at risk for infectious complications. Current antibiotic practice does not depend on the treatment phase; this permitted a fair comparison between induction and repeat therapy microbial communities and assessment of the independent effect of treatment phase. We also adjusted all our analyses for antibiotics. Our data suggest that the experience of prior intensive chemotherapy may lead to diminished microbiota stability at the time of subsequent chemotherapy, potentially resulting in greater vulnerability of microbiota to enterococcal outgrowths. While *E*. *faecalis* and *E*. *faecium* comprise up to 1% of the healthy adult gut microbiota^36^, the relative abundance of *Enterococcus* OTUs in some samples in our study approached 100%. Considering heavy antibiotic exposure in AL patients, many of the observed *Enterococcus* OTUs likely harbor antibiotic resistance genes^37^. Expansion of antibiotic-resistant enterococci during intensive therapy such as chemotherapy and hematopoietic cell transplantation (HCT) increases the risk of BSI^38,39^. Since enterococci are the third most common cause of nosocomial bacteremia in the United States with an overall mortality rate of ~30%^40^, preventing enterococcal blooms in the gut may decrease hospitalization, costs, morbidity, and mortality of curative-intent therapy in AL patients. Our sample size was not large enough to evaluate clinical outcomes.

Restoration of the gut microbiota to a healthy state can prevent and revert colonization by pathogens. Cooperating commensals, particularly obligate anaerobes, have a key role in colonization resistance to antibiotic-resistant enterococci and clearance of these pathogens after fecal microbiota transplantation (FMT)^41,42^. *Parabacteroides distasonis*, the most highly differentially abundant species in our induction therapy samples, was one of the four anaerobic commensals in the minimum consortium that successfully prevented and cleared vancomycin-resistant enterococci from the murine gut^41^. Consistent with these observations, the use of anti-anaerobic antibiotics in our cohort was associated with *Enterococcus* expansion. In addition, parenteral nutrition was a risk factor for *Enterococcus* expansion, highlighting the importance of enteral feeding, whenever possible, during intensive chemotherapy.

We suggest that microbiota restoration therapies before the initiation of repeat therapy warrant investigation. This timepoint is relevant to patients who do not achieve a remission with the first induction and those who relapse after an initial remission. Improving the stability of microbial communities before their exposure to various insults during repeat therapy may prevent pathobiont expansion and reduce infectious complications. Another timepoint where microbiota therapeutics may be beneficial is at the completion of intensive chemotherapy in patients planned to proceed to HCT. Dysbiosis has been associated with worse transplant outcomes including infections^39,43^, mortality^44–46^, graft-versus-host disease^47,48^, and relapse^49^. FMT has been safely applied after HCT, with resolution of dysbiosis^50–52^. We propose that correcting dysbiosis before HCT may be another approach to minimize infectious and non-infectious complications after HCT. Finally, as the first study on the subject, we enrolled both ALL and AML patients who received any form of intensive chemotherapy. Future studies in more uniform cohorts for disease and chemotherapy regimen are needed to evaluate whether our results are applicable to specific subgroups.

## Data Availability

Raw sequencing data are deposited under accession number SRP141394 at the NCBI SRA.
